# Say What Was Not Said

**DOI:** 10.1523/ENEURO.0128-21.2021

**Published:** 2021-04-27

**Authors:** Viktor Jirsa

**Affiliations:** Aix Marseille University, Institut National de la Santé et de la Recherche Médicale, Institut de Neurosciences des Systèmes, Marseille 13005, France

**Keywords:** Blue Brain Project, Human Brain Project, *In Silico*, model, validation

About one year ago, I learnt of the existence of *In Silico*. It did not come as a surprise, as I had expected that toward the end of the Human Brain Project (HBP)’s 10-year funding period, there will be a rush of articles and generally increased media interest in covering the project’s progress, achievements, and failures, although *In Silico* is special, given its 10-year time frame. I joined the HBP in 2014 and lead today one of three science work packages. For this documentary, I expected to hear requests for interviews, but there was literally nothing of that sort. After I have finally watched *In Silico*, I understand why. The documentary is in fact not about HBP, despite claims to the contrary by a previous review (Abbott, 2020). *In Silico* director, Noah Hutton, rather focuses on his fascination with Henry Markram’s 10-year vision of building a brain from the bottom up, the Blue Brain Project (BBP). As will be described towards the end, the HBP follows a stringent and much broader concept of integrating neuroscience and medicine with technology. In a companion commentary (Destexhe, 2021), Alain Destexhe explains that only a small percentage of the actual efforts in HBP is devoted to Henry Markram’s activities in the project, I will thus not further dwell on it.

My second expectation was to find a discussion of science, shedding light on the scientific controversy regarding the endeavor of constructing an *in silico* brain. Disappointingly, this expectation was not fulfilled either, and it is, in my opinion, a missed opportunity. Noah Hutton thematizes several points such as bottom-up construction, consciousness, as well as noise and variability. But the discussion remains shallow, hardly reaching beyond snippets of statements by neuroscientists flagging key words and reiterating the complexity of these themes.

What comes across to some degree, though, is the concern regarding the challenge of validation of BBP’s bottom-up modeling effort. Although it is never explicitly said, it is somewhat lingering in between the lines. In that context, it is suggested that emerging traveling wave activity may be seen as a first indicator of self-organization phenomena in these simulations, which have not been directly programmed into the system (see also Yves Frégnac’s companion commentary; Frégnac, 2021). This is indeed true but far from surprising. Most theoretical neuroscientists are capable of predicting these patterns and characterizing their dynamic properties analytically (that is, with paper and pencil), including velocity and wave shape, deriving them from the architecture of the network. In fact, such analysis is representative of the diverse nature and scope of the multiscale approaches harbored in HBP, linking structural and functional properties across spatial and temporal scales. HBP clearly supports bottom-up approaches typically depicting neuronal systems on microscopic scales, but it also engages macroscopic top-down approaches rooted in the mathematics of dynamical systems theory, as well as statistical physics and mean field approaches that bridge across the various scales, at which brain dynamics unfold. The multiplicity of these approaches then offers alternate routes for validation through tests of consistency and explanatory power, in relation to data, that need not be solely restricted to bottom-up modeling.

The challenge of validation is two-fold. First, there is the matter of validation of the simulated activity, emerging from the bottom-up modeling of the brain. In *In Silico*, Sebastian Seung questions whether and how we will know if a simulation truly provides the right end result. The demand is a justified request for cross validation against empirical data. A second line of response to validation addresses the link of brain activity to behavior. Zachary Mainen points out that emergent patterns of activity may or may not be meaningful and need to be linked to function. Arguably, an issue to be raised here is that behavior, in its own right, is an elusive matter which neuroscientist ultimately seek to comprehend (Frégnac, 2017; Pillai and Jirsa, 2017; Jirsa et al., 2019). I will try to unpack these points in more detail, as their substance is essentially left untouched in the documentary.

First of all, the link between a mechanistic framework for brain simulation and the resultant brain activity can be useful to understand, even in absence of a systematic behavioral interrogation, especially when it leads way to categorical classification or functional quantification that can be of practical value for diagnostics or therapeutics. For instance, markers of brain activity can be used to diagnose disease and evaluate efficiency of curative interventions. Examples of this can be found in the HBP such as the perturbational complexity index (PCI) measuring different levels of consciousness in brain states (Renzo et al., 2019), or estimates of the extent of the epileptogenic zone in patients, who are candidates for epilepsy surgery (Proix et al., 2018). The latter HBP efforts have led to the large-scale clinical trial Epinov (https://www.3ds.com/fr/recits/living-brain/) using patient-specific brain models to predict and optimize individual surgery outcome.

In these applications, variability within and between subjects can pose a daunting challenge. Yves Frégnac named it as one of the key bottleneck issues to be solved, to make a better use of Big Data in Neuroscience (Frégnac, 2017): when considering in a given brain synaptic weights for a given cell-pair type, variability in their values should not be considered as biological noise, but could, on the contrary, reflect a differential distribution of information. For a simulation to be functionally meaningful, it would then be required to retrieve information from a large range of possible realizations stored in associative memories. Simulating only the mean and the variance (devoid of information) is thus not enough and can sometimes be even wrong (Marder and Goaillard, 2006).

The same principles extend across scales, including the full brain level. When considering several brains, as each differs from each other, they maintain full functionality within a certain range of variability, but then outside of this range, brain function is diminished or lost. The functional loss, however, occurs differently for each brain. The same structural change may be functionally dramatic in one brain, but inconsequential in another. This propensity for different system configurations to support the same or similar functions is called neurodegeneracy and is at the root of the argument of many critical voices regarding mechanistic modeling in general, and the BBP in particular. Neurodegeneracy causes model non-identifiability and dilutes or destroys the concept of an optimal parameter set (as there is none). Rather there is a degenerate range of co-dependent parameters, in which healthy brain operation is possible. This is not a showstopper for bottom-up modeling approaches as in BBP, instead, it is to be conceptually integrated into the model building process. In fact, neurodegeneracy is not only possible but even necessary in the models as it is crucial for capturing the robustness of the brain against injuries and pathologies (Jirsa, 2020). As such, this criticism, alluding to an apparent challenge, is practically highlighting a research avenue ripe for scientific exploration, and thus a potential strength that would only lead to the enhancement of the resulting models.

These concepts are difficult, but we can get a handle on them through the type of approaches advocated in BBP and HBP. The link with behavior is less tangible. In the documentary it has been presented as a potential strategy (again, superficially) to validate the simulations from in silico brains. Behavior remains one of the conundrums in neuroscience, which is the more surprising as it is actually its explanandum. Said differently, we study the explanans, the brain, but are incapable of properly defining that which we actually wish to explain, the emergent behavior. As long as we are confined to summary statistics as a place holder for human behavior, we will continue to fall short in our attempts to conceptually capture and characterize behavior which is at its core a complex dynamical phenomenon. Then, naturally, we will not be able to make meaningful statements about successfully modeling a brain demonstrating behavior, because, likely, we will not even be able to recognize it if it did, given that we lack the proper language, tools, and concepts to probe for it. I intentionally exaggerate here to get the following point across: without a proper definition and operationalization of behavior, we will not be able to generalize laboratory results to more complex behaviors, and thus completely lack ecological validity.

The call for a better understanding of behavior is a theme that is being revisited regularly in neuroscience. In the 1960s and 1970s, Gibson and Bernstein were a major source of inspiration for the ecological approach of perception and action, highlighting the coordination of movement and environment. Kelso and Turvey followed in the 1980s and 1990s, attempting to quantitatively formulate the phenomenological laws underlying behavior (Kelso and Tuller, 1984). Scott Kelso, in particular, applied this dynamics perspective to explain brain mechanisms. He just recently pointed out to me that it is amusing to see articles in leading journals in the 2020s demanding more ecologically relevant behavior in neuroscience research (Krakauer et al., 2017). I agree with him. As a consequence, though, the need for a better understanding of behavior disqualifies it from being an unambiguous solid reference to be used at this point in the validation of mechanistic models of brain function, except perhaps for simple invertebrate organisms with stereotyped reflex behavior. Validation, thus, remains a big issue for the human brain. It is not a challenge specific to BBP or HBP, it is a key challenge in neuroscience at large. And this is why we need visionary scientists such as Henry Markram who break in the path and have the guts of moving forward an entire field. Terry Sejnowski stated this nicely in the documentary, that the difference between pioneers and followers is that the pioneers are the guys with the arrows in the back. Well spoken.

So *In Silico* is more a narrative about leadership than about science. Noah Hutton’s initial fascination with Markram’s 10-year vision apparently fades away as time goes by, leaving him with space for frustration at the end. Still, I would have appreciated a little more, well, appreciation of the challenges that leadership takes on at such a large-scale. It is an underrated (at least in science) but foundational factor of successful leadership: the synergetic integration across diverse domains, such as theory, experiment, technology, management, politics, communication, society, and ethics. This credo of integration resonates with and guides European funding directions, and it is not uncommon for it to manifest itself in impact statements of ambitious grant submissions. Albeit the actual execution of such integration, within a project of the complexity of a European flagship, remains a deed accomplished only by a few. It is easy to critique, write down words of wisdom and propose a novel, more sophisticated, more promising, more ethical and “whatever-better” program (see, for instance, Mainen et al., 2016). There can and should be no limits for idealism and its imagining of perfect paths. Grounded progress and impact, on the other hand, specifically in highly complex multidisciplinary domains, can only occur through arduous ventures. It needs not only be deeply rooted in excellent science but also to be framed within a clear vision, enacted as an executable uninterrupted workflow that is large enough to build societal impact, convince funders and aggregate large interest within the science community (see also Christophe Bernard's editorial addressing this point; Bernard, 2021). The HBP is a good example thereof. Since the change of leadership in the HBP, Katrin Amunts has completely reshaped the project’s organization of science and neuroinformatics efforts through the introduction of innovative integration tools such as co-design projects, show cases and voucher calls (Amunts et al., 2019), leading to the creation of a European digital research infrastructure (http://www.ebrains.eu). It is the type of work that is ultimately required to create transformative impact and leave a mark in the history of neuroscience. Ploughing through with such an ambitious workplan, regardless, or despite, of others’ skepticism toward its vision or communication style, ultimately redraws the frontiers of neuroscience by breaking new grounds. It also induces the emergence of alternative complementary approaches to address the problems that could not be solved by the original approach in the first place. This is what should happen now, maybe is already happening.

## Synthesis

Reviewing Editor: Christophe Bernard, INSERM & Institut de Neurosciences des Systèmes Decisions are customarily a result of the Reviewing Editor and the peer reviewers coming together and discussing their recommendations until a consensus is reached. When revisions are invited, a fact-based synthesis statement explaining their decision and outlining what is needed to prepare a revision will be listed below. The following reviewer(s) agreed to reveal their identity: NONE.

NA
